# A novel multigene expression construct for modification of glycerol metabolism in *Yarrowia lipolytica*

**DOI:** 10.1186/1475-2859-12-102

**Published:** 2013-11-04

**Authors:** Ewelina Celińska, Włodzimierz Grajek

**Affiliations:** 1 Department of Biotechnology and Food Microbiology, Poznan University of Life Sciences, Wojska Polskiego 48, Poznań 60-627, Poland

**Keywords:** *Yarrowia lipolytica*, Novel genetic construct, Glycerol metabolism, Heterologous expression, Biomass

## Abstract

**Background:**

High supply of raw, residual glycerol from biodiesel production plants promote the search for novel biotechnological methods of its utilization. In this study we attempted modification of glycerol catabolism in a nonconventional yeast species *Yarrowia lipolytica* through genetic engineering approach.

**Results:**

To address this, we developed a novel genetic construct which allows transferring three heterologous genes, encoding glycerol dehydratase, its reactivator and a wide-spectrum alcohol oxidoreductase under the control of glycerol-induced promoter. The three genes, tandemly arrayed in an expression cassette with a marker gene *ura3*, regulatory and targeting sequences (G3P dh promoter and XPR-like terminator, 28S rDNA as a target *locus*), were transferred into *Yarrowia lipolytica* cells. The obtained recombinant strain NCYC3825 was characterized at the molecular level and with respect to its biotechnological potential. Our experiments indicated that the novel recombinant strain stably borne one copy of the expression cassette and efficiently expressed heterologous alcohol oxidoreductase, while glycerol dehydratase and its reactivator were expressed at lower level. Comparative shake flask cultivations in glucose- and glycerol-based media demonstrated higher biomass production by the recombinant strain when glycerol was the main carbon source. During bioreactor (5 L) fed-batch cultivation in glycerol-based medium, the recombinant strain was characterized by relatively high biomass and lipids accumulation (up to 42 g_DCW_ L^-1^, and a peak value of 38%_LIPIDS_ of DCW, respectively), and production of high titers of citric acid (59 g L^-1^) and 2-phenylethanol (up to 1 g L^-1^ in shake flask cultivation), which are industrially attractive bioproducts.

**Conclusions:**

Due to heterogeneous nature of the observed alterations, we postulate that the main driving force of the modified phenotype was faster growth in glycerol-based media, triggered by modifications in the red-ox balance brought by the wide spectrum oxidoreductase. Our results demonstrate the potential multidirectional use of a novel *Yarrowia lipolytica* strain as a microbial cell factory.

## Background

Current trends in biotechnology encourage utilization of waste and renewable resources for production of high-value-added chemical compounds. High supply of raw, residual glycerol from biodiesel production plants promote the search for novel methods of its utilization [1]. In a pursue of efficient exploitation of this byproduct, multiple bioprocesses have been developed: production of 2,3-butanediol [2], 1,3-propanediol [3], vitamin B_12_[4], propionic acid [5], ethanol [6], succinic acid [7], lipids [8], citric acid [9,10] and more [11].

*Yarrowia lipolytica* is a dimorphic, nonconventional yeast species with unique metabolic properties, known for its efficient growth on raw glycerol from biodiesel production plants. Interest in this species stems from its metabolic potential expressed in exceptional ability to utilize and accumulate hydrophobic substances [12-14] as well as to produce high amounts of valuable metabolites, such as: citric and isocitric acid [15,16], succinic acid [17], erythritol [18], γ-decalactone [19] and biosurfactants [20]. *Y. lipolytica* is also frequently employed in the production of SCP and SCO from waste bioresources, such as waste cooking oil [21], agro-industrial residues [22], industrial derivatives of tallow [23], palm-oil mill or olive-oil mill wastewater [24,25]. *Y. lipolytica* is non-pathogenic for human and is considered a GRAS species, approved for numerous industrial applications [26]. This fact, together with its exceptional performance in utilization of different raw biomaterials and their bioconversion into high-value-added bioproducts stimulates its frequent application in industrial processes [27].

**Figure 1:**
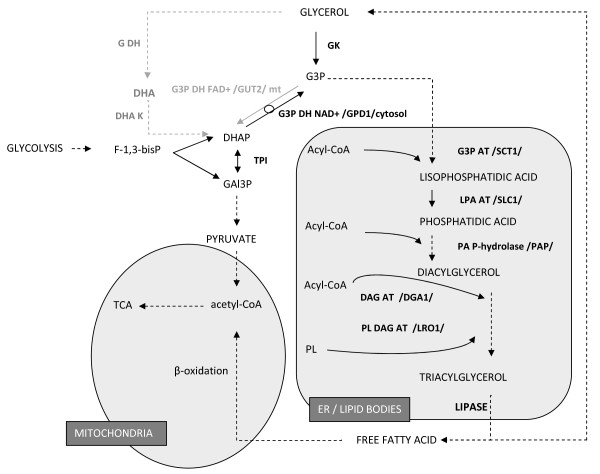
**Glycerolipids turnover in *Y. lipolytica*.** GK – glycerol kinase, G3P – glycerol-3-phosphate, G3P DH NAD+/FAD + - NAD+/FAD + -dependent dehydrogenase G3P (GPD1/GUT2 – genes encoding respective activities), DHAP – dihydroxyacetone phosphate, TPI – triosephosphate isomerase, GAl3P – glyceraldehyde-3-phosphate, F-1,3-bisP – fructose-1,3-bisphosphate, G DH –glycerol dehydrogenase, DHA – dihydroxyacetone, DHA K –DHA kinase, G3P AT – G3P acyltransferase, LPA AT, lisophosphatidic acid acyltransferase, PA P-hyrolase – lisophosphatidic acid phosphatase, DAG AT – diacylglycerol acyltranferase, PL DAG AT – phospholipids:diacylglycerols acyltransferase, PL – phospholipids. Grey dashed line – pathways inactive when grown on glycerol. Organelles are indicated by grey areas. (based on [34], [63], KEGG database; http://www.genome.jp/kegg/pathway.html).

Currently, *Y. lipolytica* constitutes a recognized system for heterologous proteins expression [28]. Direct comparison of different expression platforms: *Y. lipolytica*, *Saccharomyces cerevisiae, Pichia pastoris, Arxula adeninivorans, Hansenula polymorpha, Kluyveromyces lactis, Schizosaccharomyces pombe*[29,30] indicated that *Y. lipolytica* is characterized by several advantageous traits for heterologous proteins expression over the other expression systems. In the literature one can find several detailed review papers on the applied strategies, used vectors and heterologous proteins expressed in *Y. lipolytica*[31,32]. Apart from exploitation as a heterologous protein expression platform, efforts towards improvement of *Y. lipolytica* native metabolic properties have been pursued. Successful metabolic engineering towards increasing lipid accumulation was recently carried out using two independent approaches [33] and [13,34]. In the former report, the engineered strain was modified trough co-expression of two genes involved in triacylglycerols (TAGs) biosynthesis – diacylglycerol acyltransferase (DGA1) and acetyl-CoA carboxylase (ACC1), the final and the first activity of the pathway (see Figure 1). In the latter strategy, a deletion recombinant strain lacking all six isozymes of acyl-CoA oxidase (lack of TAGs mobilization in the stationary phase, *Δpox1-6*) and, due to disruption of the *GUT2* gene (*Δgut2*), securing higher provision of G3P (glycerol-3-phosphate) for the lipid synthesis, accumulated nearly four-fold higher amounts of total lipid per g of DCW from oleic acid [13]. Another application of metabolic engineering strategies allowed for development of a *Y. lipolytica* strain able to produce carotenoids [35,36]. This great success has been repeated and reported recently [37].

The main objective of the presented work is to develop a genetic construct bearing several heterologous genes natively involved in glycerol metabolism, and expressing the genes in glycerol-induced manner. To this end, genes encoding a vitamin B_12_-independent glycerol dehydratase *dhaB1* and its reactivator *dhaB2* from *Clostridium butyricum,* and a wide-spectrum alcohol oxidoreductase *dhaT* from *Shimwellia blattae* are cloned under a native promoter of glycerol-3-phosphate dehydrogenase (G3P dh), described as glycerol-induced [38], with the first intron sequence and strong XPR2-like terminator. Thus, the activities which are natively involved in glycerol catabolism are expressed in glycerol-induced manner.

## Results and discussion

### Construction of a novel expression integrative vector dedicated for *Y. lipolytica*

**Figure 2:**
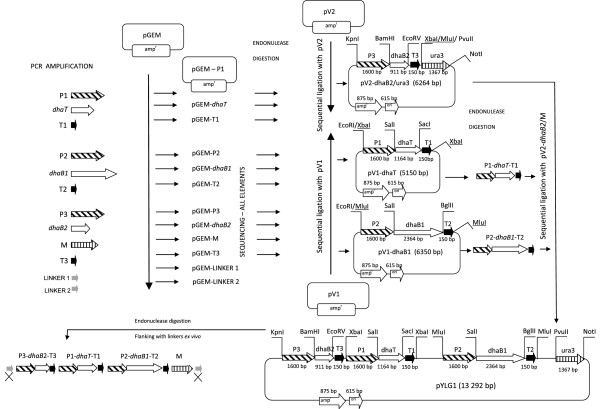
**The pYLG1 vector’s construction strategy.** Schematic representation of a strategy followed in the pYLG1 expression cassette construction. Detailed description in the text, “Materials and methods” – DNA manipulations" section.

The first key effect of the presented study is construction of a novel expression vector pYLG1 (Figure 2) bearing three heterologous genes (*dhaB1, dhaB2, dhaT*) and a selection marker gene (*ura3*). The overall length of the ready-to-transform expression cassette with *ex vivo* added flanks targeting integration at 28S rDNA *loci* is over 13 kb. The pYLG1 vector was assumed to fulfill the following requirements: transfer of heterologous genes involved in glycerol catabolism, induction of the genes expression in a glycerol-induced manner, integration of the genetic construct with the host genome and its stable bearing. These issues were addressed through the following approaches.

The pYLG1 vector presented in this work bears three heterologous genes, natively involved in glycerol metabolism. The genes *dhaB1* and *dhaB2* from *C. butyricum* encode a vitamin B_12_-independent glycerol dehydratase and its reactivator (thoroughly characterized in [39]). No such activity, catalyzing glycerol dehydratation and synthesis of 3-hyroxypropanal, has been identified in *Y. lipolytica*, thus, we decided to investigate, whether its expression in this expression system would modify its glycerol metabolism. DhaB1-DhaB2 from *C. butyricum* is the only such activity independent of vitamin B_12_ cofactor, described to date. Our preliminary experiments indicated that *Y. lipolytica* is unable to produce vitamin B_12_, and no activities involved in its production and transportation have been identified, to date (KEGG database). Therefore, glycerol dehydratase activity from *C. butyricum* was chosen for expression in *Y. lipolytica* cells. The second gene cloned in the pYLG1 vector encode a wide-spectrum alcohol oxidoreductase from *S. blattae* (formerly *Escherichia blattae*) [40]. In general, DhaT catalyses reversible reactions of oxidation and reduction between multiple small molecule aldehydes and alcohols (BRENDA; http://www.brenda-enzymes.info/). DhaT is a NAD(P)H-dependent activity contributing to the red-ox balance maintenance in a cellular system.

Each heterologous gene was flanked with regulatory elements – glycerol-induced promoter of a gene encoding G3P dh (described in [38]) and XPR2-like terminator, frequently used in different genetic constructs for *Y. lipolytica*[28,31,32]. Both the information included in [38] and own analysis of the G3P dh promoter region confirmed the presence of a spliceosomal intron in the 5’ region. According to the results presented in [38], downstream activating elements contained within this region significantly enhance expression of a reporter gene, cloned under the control of this promoter. Thus, a 1600 nt fragment including the first intron sequence was cloned in three restriction variants in the pYLG1 vector.

The parental strain used in this study is an uracil auxotroph due to integration of a *SUC2* gene from *S. cerevisiae* at the *ura3* (orotidine-5-phosphate) *locus*. The modification was completed and described in [41] with the use of pINA302 vector, generating a strain A18 with *ura3-suc2+* phenotype. Exploitation of this strain allowed selection of the obtained recombinants bearing the pYLG1 expression cassette by complementation of the auxotrophy trait with *ura3* gene contained within the vector.

The last requirement assumed for the genetic construct presented in this study was its integration with the host genome, enabling its stable bearing in the subsequent generations. It was fulfilled by flanking the expression cassette with 300 nt DNA fragments complementary to 28S rDNA gene *loci*. rDNA is a frequent target for heterologous integration due to several reasons: nucleotide sequence available for many organisms, multiple integration sites per genome, integration into a transcriptionally active site, the possibility of post-transformation amplification of the heterologous fragment’s copy number in the genome, if the selection pressure is appropriately manipulated [42-44]. In *Y. lipolytica* genome over 200 copies of rDNA clusters have been identified [45], resulting in such a number of potential integration sites.

The applied vector construction strategy (Figure 2) lead to development of the pYLG1 expression cassette used for transformation of the *Y. lipolytica* A18 competent cells.

### Characterization of the obtained recombinant *Y. lipolytica* NCYC3825 strain at the molecular level

**Figure 3:**
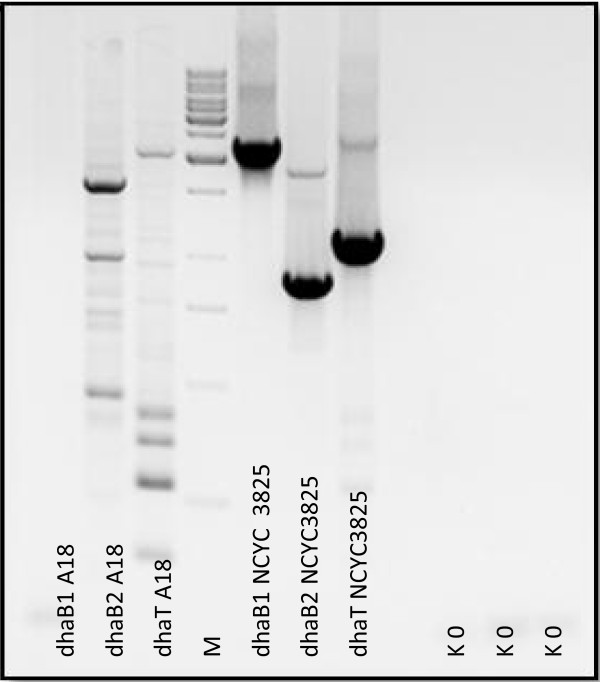
**Integration of the expression cassette with the host genomic DNA confirmed by PCR.** Agarose gel electrophoresis of amplicons obtained in PCR with primers specific to regions of heterologous genes *dhaB1, dhaB2*, and *dhaT*, respectively: 1-3: on a genomic DNA template isolated from A18 strain; 4: M: lambda-pUC19-Mix; 5-7: on a genomic DNA template isolated from NCYC3825 strain.

In a course of transformation procedure, *Y. lipolytica* NCYC3825 strain was obtained. The presence of all heterologous genes was confirmed by PCR amplification of the respective regions on the genomic DNA template (Figure 3). The obtained strain was subsequently characterized at the molecular level.

#### Copy number estimation

In order to estimate the number of copies of the expression cassette integrated with the host’s genome, a real-time PCR analysis was carried out on the genomic DNA template with a subsequent data processing according to [46]. The undertook approach allows estimation of copy number of any DNA fragment after comparison with a fragment of known number of copies. The obtained results indicated that the heterologous genes are present in one copy per genome of the recombinant strain. The final relative quantitation values obtained for *dhaB1, dhaB2*, and *dhaT* were 0.633, 0.622 and 0.627, respectively. To verify the applied method, analogous reactions were set up on the genomic DNA template of the parental strain A18. The final relative quantitation values for *dhaB1, dhaB2*, and *dhaT* were 0.017, 0.055 and 0.029, respectively. The results of this analysis confirmed that the sequences were absent from the parental strain’s genome. Presence of a single copy of the expression cassette was rather unexpected, since 28S rDNA was targeted for integration. As mentioned earlier, over 200 copies of rDNA cluster were identified in *Y. lipolytica* genome. Thus, integration of higher number of expression cassette copies was expected. Two possible reasons could contribute to such a result: short fragments of homology used as targeting sequences and low quantity of the expression cassette used for transformation. According to [43], 300 nt is the minimal length of homology regions allowing integration occurrence, however, usually whole homologous genes, endonuclease digested in a central region, were used as the targeting elements. On the other hand, our experience show that even much shorter homology regions (60 nt) are sufficient for occurring an integration event.

#### Stability assessment

In the following step, stability of the expression cassette in the recombinant’s genome was assessed through PCR amplification of the heterologous genes’ regions on the genomic DNA template. Stability of the expression cassette was assessed in the following conditions: multiple (>40 times) passaging in the MMT agar selective medium, lyophilization followed by reviving process, and bioreactor culturing, when no selection pressure was maintained (uracil was contained in the medium). The obtained images of electrophoretic separation of amplicons indicated that during all of the applied conditions, the expression cassette was stably maintained in the host’s genome.

#### Gene expression analysis

**Figure 4:**
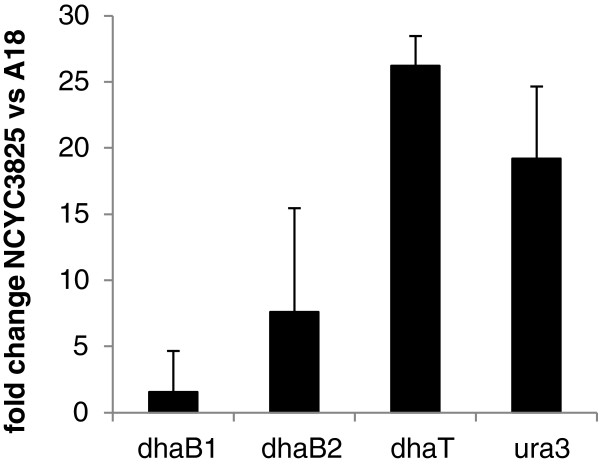
**Heterologous genes expression level.** Heterologous genes expression level determined through real-time PCR analysis, expressed as fold change in the NCYC3825 strain, normalized *vs* the A18 strain. Data were processed according to ΔΔC_T_ method [73]. All the samples were analyzed in triplicate. Error bars indicate SD.

Expression of the heterologous genes in the novel expression system was analyzed through real-time quantitative PCR performed on the cDNA template obtained from the parental A18 and the recombinant NCYC3825 strain. The results expressed as fold change of the normalized expression level in the recombinant strain cells *vs* the parental strain cells are presented in (Figure 4). No amplification products were obtained in the reaction with primers specific to XPR2-like terminator, indicating lack of genomic DNA contamination. As shown in (Figure 4) the expression level of clostridial genes, *dhaB1* (1.56-fold increase) and *dhaB2* (7.63-fold increase), is relatively low, when compared to the expression level of *dhaT* (26.2-fold increase) gene; *ura3* gene was analyzed as a positive control (19.2-fold increase). Since all the heterologous genes were cloned under the control of the same G3P dh promoter, the differences in the expression level should not result from differences in a number of transcription initiation events. Analysis of DNA sequences of the heterologous genes showed considerable differences in GC content, which for clostridial genes equaled 29%, for *dhaT* - 59%, and 50% for *Y. lipolytica* genomic DNA. According to [47-49] significant differences in GC content between heterologous sequence and sequences native to the host genome can contribute to low expression efficiency of the heterologous sequence, caused by premature transcription termination or improper maturation of mRNA. It is highly probable that the difference in GC content between *Y. lipolytica* genome and the clostridial genes contributed to lower expression efficiency. Codon usage optimization of the clostridial sequences could probably improve the expression efficiency. Such an approach was shown to improve the expression efficiency, when the donor of the expressed sequence and the host organisms were phylogenetically distant [50-52].

#### Oxidoreductase activity in a new expression system

**Figure 5:**
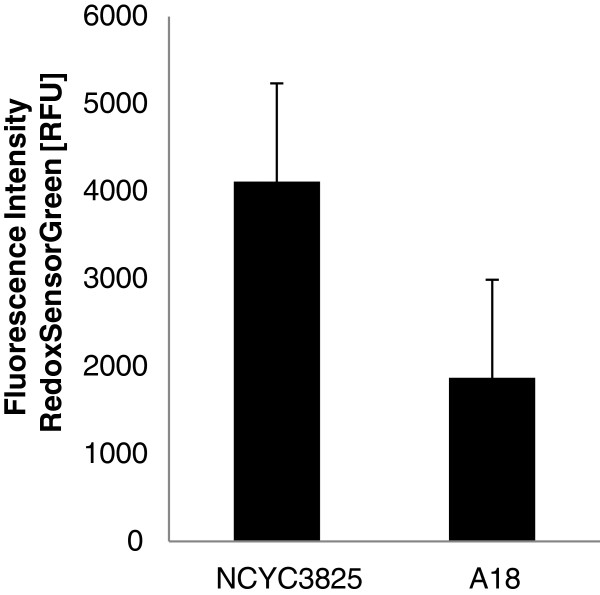
**Comparative determination of the overall oxidoreductase activity in NCYC3825 and A18 cells.** Comparative determination of the overall oxidoreductase activity in the recombinant NCYC3825 and the parental A18 strains expressed as fluorescence intensity, determined after staining with BacLight™ Redox Sensor Green Vitality Kit (Life Technologies, Invitrogen) oxidoreductase activity sensor, followed by flow cytometry analysis. RFU – relative fluorescence units. Statistical importance of the observed difference was analyzed with t-Student’s test (MS Excel AnalysisToolPack) at p < 0.001. Error bars indicate SE.

Since DhaT cloned and expressed in *Y. lipolytica* NCYC3825 catalyzes multiple NAD(P)H-dependent reactions of oxidation and reduction between various small molecular weight alcohols and aldehydes, the overall oxidoreductase activity assessment in the parental and the recombinant strain cells was carried out. To this end, flourophore staining and cytometric analysis were used. Results of this analysis are shown in (Figure 5). The recombinant NCYC3825 strain was characterized by over two-fold higher oxidoreductase activity when compared to the parental strain A18 (p < 0.001; t-Student’s test). Such results could be attributed to heterologous activity of the DhaT oxidoreductase, however, also different physiological properties of the two compared strains could influence the obtained result, like more vigorous growth of the NCYC3825 prototroph.

The observed characteristics of the NCYC3825 strain were similar to those obtained in the *VHb+ Y. lipolytica* strain [53], where a gene encoding hemoglobin from *Vitreoscilla* was expressed. Both strains were characterized by higher growth rate and oxygen consumption, and by higher production of extracellular proteins. Multiple enzymatic activities and cellular processes contribute to development of these characteristics, thus it seems that a more general molecular process was modified in both of these strains. The expressed heterologous proteins posses different molecular roles, NAD(P)H-dependent red-ox reactions (DhaT) and intracellular oxygen supply (VHb), however, both are involved in the red-ox cofactor turnover. In the face of the obtained result regarding oxidoreductase activity in the NCYC3825 strain and the macroscopic characteristics of both, NCYC3825 and *VHb+,* strains, it can be postulated that modification of the red-ox potential mainly contributed to the observed differences between A18 and NCYC3825 strains. This statement is corroborated by the other literature data, where *VHb* gene was heterologously expressed and consequently lead to improvement of the modified strains’ performance during cultivation [54-57].

### Performance of *Y. lipolytica* NCYC3825 recombinant strain during cultivation

In this series of experiments the novel recombinant strain NCYC3825 was directly compared with its parental strain A18. Direct comparison of these strains is not fully justified, due to different metabolic background of these strains. Obviously even supplementation of the culturing media for the auxotroph with uracil does not fully account for its deficiency. Nevertheless, the observed characteristics of here obtained recombinant strain compares favorably with the literature data, illustrating the strain’s biotechnological potential as a multidirectional microbial cell factory.

#### Biomass growth and glycerol utilization

**Figure 6:**
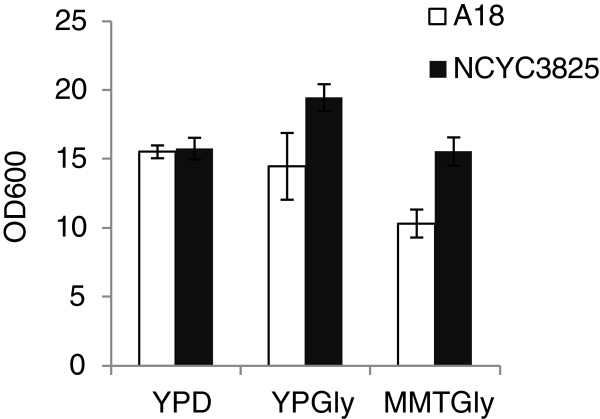
**Biomass production by NCYC3825 and A18 strains in YPD, YPGly and MMTGly media.** Final biomass yield (OD at 600 nm wavelength) was determined at the end of shake flask cultivations. Experimental conditions are described in “Materials and methods - Microbial strains, media and culture conditions” section. The experiment was carried out in triplicate. The observed differences were statistically important at p < 0.05 (analyzed with t-Student’s test (MS Excel AnalysisToolPack)). Error bars indicate SD.

**Figure 7:**
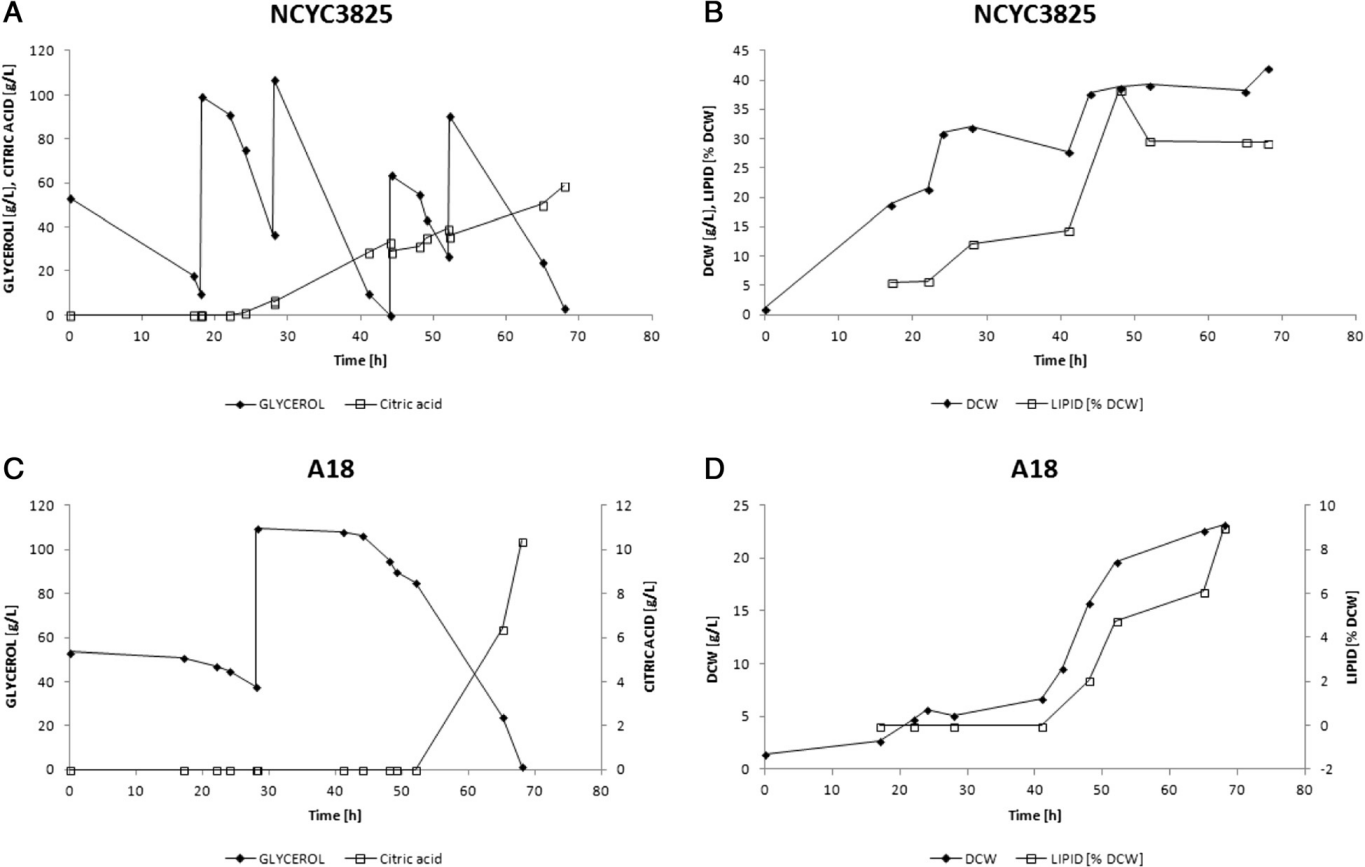
**Fed-batch bioreactor cultivation of NCYC3825 (A.B.) and A18 (C.D.) strains in a glycerol-based media.** Representative kinetics of glycerol utilization (g L^-1^) and citric acid production (g L^-1^) **(A.C.)**, biomass formation (DCW, g L^-1^) and percentage of total lipid fraction in DCW (% DCW) **(B.D.)** of the recombinant *Y. lipolytica* NCYC3825 strain **(A.B.)** and the parental strain A18 **(C.D.)** during bioreactor fed-batch cultivation in a glycerol-based medium. Experimental conditions are described in “Materials and methods - Microbial strains, media and culture conditions” section, and quantitative summary is provided in (Table 1).

Preliminary shake flask cultivations in YPD, YPGly and MMTGly media indicated that the recombinant NCYC3825 strain exhibits higher biomass formation than the parental strain A18 when glycerol was the main carbon source (YPGly and MMTGly media; p < 0.05, t-Student’s test) (Figure 6). No significant differences in the biomass yield were observed in YPD medium (p < 0.05, t-Student’s test). These results were further verified in bioreactor cultivations with glycerol as the main carbon source. The cultivation was carried out in a fed-batch mode, and the time of feeding of a new medium portion was dictated by glycerol utilization level (Figure 7). Since the recombinant strain grew more rapidly than the parental strain, four portions of the feed were added into the recombinant’s culture, while only one portion of the feed was fed into the parental strain’s culture, during the same process time. The final biomass yield and productivity were of 42 g L^-1^ and 0.62 g L^-1^ h^-1^ for the recombinant strain and 23 g L^-1^ and 0.34 g L^-1^ h^-1^ for the parental strain (Table 1). The reason behind increased biomass formation could be attributed to modified red-ox potential caused by DhaT action. According to the literature data, altered balance in reducing equivalents turnover, triggered by either oxidoreductase or *VHb* gene expression, brought similar macroscopic characteristics of the modified yeast strains, marked by *i.a.* improved biomass formation [58-60].

**Table 1:** Quantitative comparison of the results obtained in bioreactor fed-batch cultivation in a glycerol-based medium

	**DCW**	**DCW**	**CA**	**CA**	**CA**	**CA**	**LIPIDS**	**LIPIDS**	**LIPIDS**
	**[g L**^**-1**^**]**	**[g L**^**-1**^ **h**^**-1**^**]**	**[g L**^**-1**^**]**	**[g L**^**-1**^ **h**^**-1**^**]**	**[g g**_**DCW**_ ^**-1**^**]**	**[g g**_**glycerol**_ ^**-1**^**]**	**[g L**^**-1**^**]**	**[g g**_**DCW**_ ^**-1**^**]**	**[g g**_**glycerol**_ ^**-1**^**]**
**NCYC3825**	42	0.62	58.8	0.86	0.89	0.17	13	0.3	0.039
**A18**	23	0.34	10.4	0.15	0.32	0.08	2.98	0.09	0.024

DCW – dry cellular weight, CA – citric acid, LIPIDS – total lipids. Final titer [g L^-1^], volumetric productivity [g L^-1^ h^-1^], biomass-normalized titer [g OD_600_ ^-1^] or [g g_DCW_ ^-1^], yield [g g_glycerol_ ^-1^]. Statistical importance of the observed differences was analyzed with t-Student’s test (MS Excel AnalysisToolPack) – the observed differences were statistically important at p < 0.05. For kinetics presentation see (Figure 7).

#### Production of industrially attractive metabolites

The final stage of the presented study was metabolic characterization of the obtained recombinant strain, with a particular focus on production of industrially attractive metabolites. To this end, culture supernatants were analyzed by GC/MS and HPLC. Furthermore, since one of the most interesting traits of *Y. lipolytica* is its ability to accumulate high amounts of lipids, cellular lipid content and fatty acid profiles were determined. These analyses are relevant with respect to the ultimate aim of the presented study (modification of glycerol catabolism), since, after entering the cell, glycerol is phosphorylated to G3P which is the second substrate (with acyl-CoA) for TAGs biosynthesis (compare (Figure 1). The level of intracellular G3P was shown to significantly influence TAGs accumulation [34].

The recombinant strain produced considerably higher amounts of citric acid, when compared to the parental strain, reaching respectively: 58.8 *vs* 10 g L^-1^ of citric acid, with volumetric productivity 0.86 *vs* 0.15 g L^-1^ h^-1^ and yield 0.17 *vs* 0.07 g g^-1^ (Table 1). The obtained final titer of citric acid constitutes an interesting result compared to the literature data, but obviously requiring further optimization. One of the earliest paper on glycerol bioconversion into citric acid using *Y. lipolytica* reported the final titer of citric acid reaching 35 g L^-1^[61]. Further studies brought successive improvement up to 62.5 g L^-1^ in flask cultures during 600 h [11]. Most recently, advanced cultivation approaches and/or selection of mutagenized strains regarding the ability to produce higher titers of citric acid lead to outstanding achievements. Repeated batch cultivation allowed for improvement of citric acid final titers from 112 g L^-1^ to 124 g L^-1^[9] when A-101-1.22 strain was grown in a waste glycerol-based medium. In another paper by the same research team, strains *Y. lipolytica* Wratislavia AWG7 and Wratislavia 1.31 produced up to 157.5 g L^-1^ of citric acid in a fed-batch culture from waste glycerol [62]. N1 mutant strain, generated through random chemical mutagenesis, produced 217 g L^-1^ of citric acid when grown on petrolatum, and 120 g L^-1^ of citric acid when ethanol was used as a carbon source [15]. Another mutant strain by this research team, *Y. lipolytica* N15, was tested in media containing pure and waste glycerol, producing 98 and 71 g L^-1^ of citric acid, respectively [10].

Analysis of the lipid content in NCYC3825 and A18 cells grown in a glycerol-based medium showed superior ability of the former strain to accumulate significant amounts of lipids under applied culturing conditions (Figure 7). The ultimate values of lipid content in the culture and the percentage of lipid fraction in DCW at the end of culturing reached 13 *vs* 3 g L^-1^ and 29.3% (with a peak value of nearly 40% of total lipid content in DCW) *vs* 9.03% for the recombinant and the parental strain, respectively. Interpretation of these results should be carried out while bearing in mind that growth of some *Y. lipolytica* strains in glycerol-based media is divided into three growth phases: biomass formation, lipid accumulation and citric acid production [12,63]. Then, it becomes obvious that the principal trait of the recombinant strain distinguishing it from the parental strain is its faster growth. The observed higher accumulation of lipids and higher titers of citric acid are related to earlier entering “lipogenic” and subsequent switching to “citric acid production” phases. In [63] it has been postulated that lipid turnover, concomitant with entering citric acid production phase, is related to reduced activity of glycerol kinase in the stationary phase of growth, when the glycerol uptake rate is reduced and its intracellular concentration is low. Then, storage lipids are mobilized and used for maintenance purposes.

The results obtained in the presented study with NCYC3825 strain concerning lipid synthesis per g of DCW (~40% of DCW) are comparable with the best values found in the literature, while the final titer of lipids per culture volume (13 g L^-1^) represents one of the best results reported to date. Corresponding results with respect to lipid fraction in DCW were obtained with *Y. lipolytica* ACA-YC 5033 strain (2 g L^-1^, 30% of DCW, even though the culture was carried out in shake flasks) [64] and *Y. lipolytica* LGAM S(7)1 (3.5 g L^-1^, 43% of DCW) when cultured in continuous mode on raw-glycerol-based medium [8]. 30% of DCW was represented by lipids in *Y. lipolytica* N15 mutant grown on crude glycerol during a biomass formation phase (~ 6 g L^-1^), with a sharp decrease in lipid content after entering citric acid production phase [10]. Repeated batch mode cultivation in pure-glycerol-based medium allowed for maximal storage lipid accumulation reaching over 20% of DCW (1.3 g L^-1^) in *Y. lipolytica* ACA-DC 50109 cells, followed by a nearly two-fold decrease in the subsequent growth phase [63]. The observed high lipid fraction in *Y. lipolytica* NCYC3825 could be attributed to fast growth rate concomitant with prolonged growth phase due to fed-batch mode of cultivation, which is one of the strategies aiming at improvement of microbial oil production. Different strategy towards increasing storage lipid accumulation was applied and described in [65], where after carbon source exhaustion and reaching stationary phase of growth, acetic acid was added into the culture, as a substrate for bioconversion into lipids. Such approach lead to accumulation of lipids at 40% of DCW. The highest level of *de novo* synthesized lipids from non-hydrophobic substrate was obtained by genetically engineered strain, which accumulated 41.4% of DCW as lipids in shake flask cultivations, and 61.7% of DCW in bioreactor cultivation (120 h) on glucose [33]. The engineered strain was modified trough co-expression of two genes involved in triacylglycerols (TAGs) biosynthesis – diacylglycerol acyltransferase (DGA1) and acetyl-CoA carboxylase (ACC1), the final and the first activity of the pathway (see Figure 1). Such approach, increased rate of the first (“push” the carbon flux) and the last (“pull” the carbon flux) reactions of the lipid synthesis pathway, leading to higher total lipid accumulation per g of DCW.

*Ex novo* storage lipid accumulation reached 54% and 33% of DCW when *Y. lipolytica* was grown in a batch mode on industrial fat as the sole carbon source and on a mixture of industrial fat and glycerol, respectively [22,66]. In [23], relatively high accumulation of intra-cellular lipid was reported, when *Y. lipolytica* ACA-DC 50109 was grown in the presence of solid industrial derivative of tallow in shake flasks (53% of DCW), and lower, when the culture was carried out in the bioreactor (up to 16% of DCW). The authors suggested that such a discrepancy between slightly aerated flask cultures and highly aerated bioreactor cultures triggers differences in activity of acyl-CoA oxidases, higher at greater oxygen provision. Considering this statement, the results obtained with the NCYC3825 strain are particularly interesting, as such high level (40% and 13 g L^-1^) of lipid synthesis was achieved in highly aerated and stirred culture. On the other hand, the report [33] indicated the opposite trend – higher lipid accumulation was observed in the bioreactor culture. The highest value of *ex novo* storage lipid accumulation, 70% of DCW, was reported for genetically engineered strain lacking G3P dh activity (*Δgut2*; higher provision of G3P for lipid synthesis) cultured in the presence of ultrapure oleic acid [13]. In the same report a “double” knock-out recombinant strain (*Δgut2Δpox1-6* = lack of unproductive G3P dissipation and lack of stored lipid mobilization) was characterized. The strain accumulated 13% and 42% of its DCW as lipid, when grown in glucose- and oleic acid-based medium, respectively. This comparison illustrates the strain’s potentialities in *de novo* and *ex novo* lipid accumulation, with significant advantage of the latter. Comparable results regarding *ex novo* lipid synthesis were reported for genetically engineered strains over-expressing *GPD1* gene in *Δpox1-6* and *Δgut2Δpox1-6* background, accumulating 65% and 70% of DCW as lipid, respectively, when grown in oleic acid-based medium [34].

Recently, successful metabolic engineering approaches towards modification of fatty acid composition in *Y. lipolytica* have been reported. Metabolic engineering strategy allowed development of *Y. lipolytica* strain producing eicosapentaenoic acid at high level [67] or increased accumulation of conjugated linoleic acid, known for its salutary properties [68]. Fatty acid composition analyses (Table 2) of the total lipid fraction in NCYC3825 and A18 cells at the end of bioreactor cultivation (Figure 7) showed that oleic acid was the main component of lipids, followed by palmitic and linoleic acids, which is commonly observed in various oleaginous fungi [for comprehensive study on fatty acid profile in oleaginous fungi see [69]). As postulated here, NCYC3825 and A18 cells were at different metabolic phase at the time of sample collection. While NCYC3825 already entered citric acid production phase, A18 cells were at late biomass formation phase. Therefore, A18 total lipid samples could represent characteristic profile of fatty acids at the onset of lipogenesis, while NCYC3825 samples, could serve as representative samples of citric acid production phase, since no direct modification of glycerolipids turnover was introduced to the recombinant strain. The most significant change in fatty acid profile between the two samples is abrupt drop (by nearly 10%) in oleic acid abundance in NCYC3825 cells. These results greatly correspond with those by [63], where significant decrease in oleic acid contribution to the total lipid fraction was observed for *Y. lipolytica* cells after entering citric acid production phase. Such observation may suggest that this fatty acid is rapidly mobilized from storage lipids along with entering citric acid production phase.

**Table 2:** Fatty acid composition profiles at the end of bioreactor cultivation in glycerol-based media

**Fatty acid**	**A18**	**NCYC3825**
Myristic acid (C14:0)	0,30	0,21
Pentadecanoic acid (C15:0)	0,24	0,41
Palmitic acid (C16:0)	12,56	15,38
Palmitoleic acid (C16:1n9)	0,77	2,79
Palmitoleic acid (C16:1n7)	9,86	11,10
Heptadecanoic acid (C17:0)	0,14	0,17
cis-10-Heptadecanoic acid (C17:1)	0,84	0,82
Stearic acid (C18:0)	4,54	4,20
Oleic acid (C18: 1n9c)	47,48	38,88
Oleic acid (C18: 1c11)	1,64	1,92
Oleic acid (C18:1c15)	0,08	0,26
Linoleic acid (C18:2n6c)	13,26	14,58
Linoleic acid (C18:2c9c15)	5,79	7,50
α-Linolenic acid (18:3 n3)	0,31	0,59
Behenic acid (C22:0)	0,00	0,07
Erucic acid (C22:1n9)	0,25	0,22
Arachidonic acid (C20:4n6)	0,00	0,03
cis-13,16-Docosadienoic acid (C22:2)	0,08	0,02
cis-5,8,11,14,17-Eicosapentanoic acid (C20:5n3)	1,89	0,85
**Total of fatty acids: Saturated**	17,76	20,44
**Total of fatty acids: Monounsaturated**	60,91	55,98
**Total of fatty acids: Polyunsaturated**	21,32	23,57

The numbers provide mean percentage content of total lipid fraction represented by the respective fatty acid. The analyses were carried out in duplicate.

**Figure 8:**
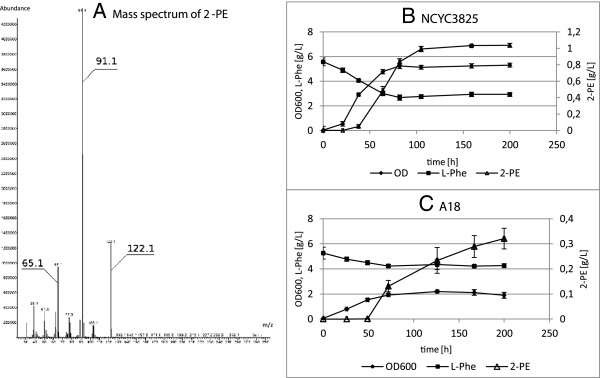
**2-Phenylethanol production by NCYC3825 and A18 strains in a glycerol-based medium. A**. Mass spectrum of 2-PE which allowed identification of the compound, obtained after GC/MS analysis. (**B**. NCYC3825, **C.** A18) Kinetics of 2-Phenylethanol production, L-Phenylalanine utilization and biomass formation (OD at 600 nm wavelength) during shake flask cultivations in a glycerol-based medium. Experimental conditions are described in “Materials and methods - Microbial strains, media and culture conditions” section, and quantitative summary is provided in (Table 3). The experiment was carried out in triplicate. Error bars indicate SD.

Finally, GC/MS analysis of culture supernatants showed that the novel recombinant strain NCYC3825 produces considerable amounts of 2-phenylethanol (2-PE), which is a valuable aroma compound (US$ 1000 per kg), widely used in food and cosmetics production. The ability to produce 2-PE by *Y. lipolytica* strains, has not been described for *Y. lipolytica* species previously (for more details on 2-PE production by *Y. lipolytica* please refer to [70]). The recombinant strain produced significantly higher titers of 2-PE than the parental strain (1 g L^-1^*vs* 320 mg L^-1^; statistical importance at p < 0.05, t-Student’s test) under applied conditions - in shake flask cultivation with glycerol as a carbon source (Figure 8, Table 3). The exact course of the 2-PE biosynthesis pathway in *Y. lipolytica* remains to be elucidated, however, it appears that it follows the general routes of L-Phe bioconversion (Ehrlich pathway), as supplementation with this amino acid strongly increases 2-PE production.

**Table 3:** Quantitative comparison of cultivation parameters obtained in shake flask cultivations aiming at 2-PE production

	**2-PE**	**2-PE**	**Utilized L-Phe**	**2-PE/L-Phe**	**2-PE/biomass**	**OD**_**600**_
	**[mg L**^**-1**^**]**	**[mg L**^**-1**^ **h**^**-1**^**]**	**[g L**^**-1**^**]**	**[g**_**2-PE **_**g**_**L-Phe**_ ^**-1**^**]**	**[g OD**_**600**_ ^**-1**^**]**	**[OD**_**600 **_**units]**
**NCYC3825**	1056.0	4.7	2.57	0.41	0.19	5.29
**A18**	320.6	1.68	0.99	0.32	0.17	1.88

Final titer [mg L^-1^], volumetric productivity [mg L^-1^ h^-1^], utilized L-Phe [g L^-1^], yield 2-PE from L-Phe [g_2-PE_ g_L-Phe_ ^-1^], biomass normalized 2-PE titer 2-PE/biomass [g OD_600_ ^-1^], biomass production expressed as turbidity at 600 nm wavelength [OD_600_ units]. Experiments were carried out in triplicate. Statistical importance of the observed difference was analyzed with t-Student’s test (MS Excel AnalysisToolPack) – the observed differences were statistically important at p < 0.05. For kinetics presentation see (Figure 8).

With respect to the genetic modification introduced to NCYC3825 strain, an increase in 2-PE biosynthesis can be primarily attributed to faster growth of the recombinant strain (greater biomass production), which is perfectly illustrated by the biomass-normalized productivity parameter, 0.19 vs 0.17 [g OD_600_^-1^] for the recombinant and the parental strain, respectively (lack of difference in this parameter between the two strains was confirmed by t-Student’s test at p < 0.05). 2-PE production in the yeast cells is predominantly a bioconversion process (L-Phe converted into 2-PE), and contribution of *de novo* synthesis, through the shikimate pathway, is rather negligible [71]. Therefore, the more cells per volume, the more “catalyst” per volume, leading to higher titers of the product. Another, less probable, hypothesis explaining superior production of 2-PE by the NCYC3825 strain could be higher provision of reducing equivalents obtained in the recombinant cells, which are required for the final reaction of 2-PE formation. The enzymatic activity of phenylacetaldehyde dehydrogenase (PhAcAld dh), catalyzing the final reduction step in the 2-PE formation, has not been identified to date. For the other yeast species PhAcAld dh has been postulated to be NADH-dependent alcohol dehydrogenase. Assuming PhAcAld dh being the bottleneck of the 2-PE formation, significant increase in oxidoreductase activity providing higher abundance of reducing equivalents could importantly contribute to an increase in 2-PE synthesis. However, these considerations remain highly hypothetical and implausible.

## Conclusions

In this study we attempted modification of glycerol catabolism in *Yarrowia lipolytica* strain through genetic engineering approach. To this end, we designed and constructed a novel integrative expression vector bearing three heterologous genes, natively involved in glycerol metabolism and controlled in glycerol-induced manner by G3P dh promoter. We proved that the vector can be stably integrated with the host genome in operational manner. Expression of wide-spectrum alcohol oxidoreductase was driven at high level, while expression of clostridial genes was maintained at lower level. Here obtained recombinant strain was characterized by several interesting traits: relatively high biomass formation (42 g L^-1^) and lipids accumulation (peak value 38%_LIPIDS_ of DCW), production of citric acid (58 g L^-1^) and 2-phenylethanol (1 g L^-1^) in glycerol-based media. Due to heterogeneous nature of the observed alterations, we postulated that the main driving force of the modified phenotype was faster growth of the recombinant strain, triggered by modification in the red-ox potential. Our results demonstrate the potential multidirectional use of a novel *Yarrowia lipolytica* strain as a microbial cell factory.

## Materials and methods

### Microbial strains, media and culture conditions

*Y. lipolytica* A18 and A10 strains used in this study were kindly donated by Prof. M. Robak from Department of Biotechnology and Food Microbiology, Wroclaw University of Environmental and Life Sciences, Poland. *S. blattae* and *C. butyricum* strains were purchased from ATCC and DSMZ collections, respectively, and were handled according to the protocol provided by the supplier. The obtained recombinant strain, *Y. lipolytica* NCYC3825, has been deposited for a patent purpose in the National Collection of Yeast Cultures (Norwich, UK). Strains used in this study are summarized in (Table 4).

**Table 4:** Microbial strains and vectors used in this study

**Strain**	**Relevant characteristics and application**	
*Yarrowia lipolytica* A18	*ura3-suc2+*	Host strain
*Yarrowia lipolytica* A10	wild type	Natural isolate, donor of regulatory elements for expression cassette
*Clostridium butyricum* DSM10702	wild type	Donor of *dhaB1* and *dhaB2* genes sequences
*Shimwellia blattae* ATCC334030	wild type	Donor of a *dhaT* gene sequence
*Escherichia coli* JM109	*endA1 glnV44 thi-1 relA1 gyrA96 recA1 mcrB*^*+*^*Δ(lac-proAB) e14- [F' traD36 proAB*^*+*^*lacI*^*q*^*lacZΔM15] hsdR17(r*_*K*_^*-*^*m*_*K*_^*+*^*)*	Laboratory strain used as a host for routine cloning of pGEM-T-Easy constructs and intermediate constructs of the final vector
*Yarrowia lipolytica* NCYC 3825	*Δura3 + suc2 + dhaB1 + dhaB2 + dhaT+*	The recombinant strain obtained in this work, deposited in NCYC, Norwich, UK
**vectors and genetic constructs**		
*pGEM-T-Easy/Promega*	T/A amplicons cloning	
*pBR322/Life Technologies*	donor of ori and β-lactamase	
*pV1/this work*	pBR322-based construction platform, circularized with linker 1	
*pV2/this work*	pBR322-based construction platform, circularized with linker 2	
*pV1-dhaT/this work*	*EcoRI/XbaI---PROM G3Pdh I---SalI---dhaT---SacI---TER XPR2-like I---XbaI -- ampr—ori--*	
*pV1-dhaB1/this work*	*EcoRI/MluI---PROM G3Pdh 2---SalI---dhaB1---BglII---TER XPR2-like 2---MluI -- ampr—ori--*	
*pV2-dhaB2/ura3d/this work*	*KpnI--PROM G3Pdh 3--BamHI--dhaB2--EcoRV--TER XPR2-like 3--XbaI--PvuII--ura3-NotI*--*ampr—ori--*	
*pYLG1/this work*	*KpnI--PROM G3Pdh 3--BamHI--dhaB2--EcoRV--TER XPR2-like 3-- XbaI---PROM G3Pdh I---SalI---dhaT---SacI---TER XPR2-like I---XbaI -- MluI---PROM G3Pdh 2---SalI---dhaB1---BglII---TER XPR2-like 2---MluI PvuII--ura3-NotI—amp*^*r*^*—ori--*	
*Expression cassette/this work*	*rDNA R--KpnI--PROM G3Pdh 3--BamHI--dhaB2--EcoRV--TER XPR2-like 3-- XbaI---PROM G3Pdh I---SalI---dhaT---SacI---TER XPR2-like I---XbaI -- MluI---PROM G3Pdh 2---SalI---dhaB1---BglII---TER XPR2-like 2---MluI PvuII--ura3-NotI-rDNA L*	

Routine laboratory cultivation of *E. coli* JM109 parental strain, as well as all the following transformants was carried out according to the standard laboratory procedures [72]. Typically *E. coli* strains were cultured in LB medium, containing (per liter): glucose 10 g, Bacto Peptone 10 g, yeast extract 5 g. For transformants cultivation ampicilin was added (at 100 mg L^-1^) to maintain selective pressure. *Y. lipolytica* A18, A10, and the obtained recombinant NCYC3825 strains were cultured in YPD or MMT and maintained according to the protocols described in [28]. Typically the yeast strains were cultured at 30°C, 250 rpm on a rotary shaker in YPD medium, containing per liter: Bacto Peptone 20 g, yeast extract 10 g, glucose 20 g. Alternatively yeast strains were routinely cultured in MMT medium, containing per liter: NH_4_H_2_PO_4_ 5 g, KH_2_PO_4_ 2.5 g, MgSO_4_x7H_2_O 1 g, glucose 10 g, thiamine 3 mg, trace elements solution II 1.0 mL per liter of deionized water. Minimal media for the parental auxotroph strain were supplemented with uracil at 2 mg L^-1^.

Preliminary cultivation tests were carried out in shake flasks in total culture volume of 20 mL. Rich (YPD) medium was prepared as described in the preceding paragraph. YPGly was prepared as follows: Bacto Peptone 20 g, yeast extract 10 g, glycerol 20 mL per liter of deionized water. MMTGly, modified by substitution of glucose with glycerol, [28] contained: NH_4_H_2_PO_4_ 5 g, KH_2_PO_4_ 2.5 g, MgSO_4_x7H_2_O 1 g, glycerol 10 g, thiamine 3 mg, trace elements solution II 1.0 mL per liter of deionized water. Cultures of the parental and the recombinant strains were carried out in triplicate, at 30°C, with shaking at 250 rpm.

Shake flask cultivations of NCYC3825 and A18 yeast strains aiming at 2-PE production from L-Phe was carried out in glycerol-based medium, containing per liter: glycerol 50 g, KH_2_PO_4_ 15 g, MgSO_4_x7H_2_O 0.5 g, Yeast Nitrogen Base 0.2 g, L-phenylalanine 5 g, trace elements solution II 1.0 mL, thiamine 3 mg. Cultures of the parental and the recombinant strains were carried out in triplicate, at 30°C, with shaking at 250 rpm. Time point samples were stored at -20°C for subsequent liquid analysis (HPLC) and biomass growth determination (OD_600_).

Bioreactor cultivations were carried out in BIOSTAT® A plus (Sartorius) stirred-tank bioreactors, total volume of 5 L, culture medium volume of 2 L. Culture medium was inoculated with 10% (v/v) 48 h YPD culture. pH and temperature were automatically adjusted to 4.0 and 30°C. Stirring and aeration were automatically adjusted to maintain oxygen saturation of the culture at 30%. Culture parameters were automatically monitored with BioPAT® MFCS SCADA. Fed-batch culture aiming at maximization of biomass formation was carried out in medium containing: glycerol 40 mL, (NH_4_)_2_SO_4_ 6 g, KH_2_PO_4_ 1.1 g, MgSO_4_x7H_2_O 0.14 g, yeast extract 10 g, Bacto pepton 0.5 g, trace elements solution II 1.0 mL, AntiFoam 204 (Sigma-Aldrich) 0.5 mL per liter of deionized water (plus external supplementation with AntiFoam 204 during cultivation). The bioreactor volume was completed with 0.5 kg of the feed medium, containing: glycerol 390 mL, (NH_4_)_2_SO_4_ 60 g, yeast extract 10 g per liter of deionized water; dosing of the feed depended on the measured glycerol concentration in the culture. Time point samples were stored at -20°C for subsequent liquid analysis (HPLC) and biomass growth determination (gravimetrically).

### Molecular biology tools and protocols

#### Competent cells and transformation

Preparation of *E. coli* CaCl_2_/MgCl_2_ competent cells and heat-shock transformation were carried out according to the standard molecular biology protocols [72]. Preparation of *Y. lipolytica* A18 competent cells and transformation *via* a lithium acetate procedure were completed according to [28] protocols, and stocks of the prepared competent cells were liquid nitrogen-frozen and stored at -80°C. Before transformation, the competent cells were thawed on ice and washed twice in lithium acetate buffer, according to the applied protocol.

#### DNA manipulations

Standard molecular biology techniques were used throughout this study [72]. Vectors used in this study are summarized in (Table 4). Primers and linkers used in this study are listed in Additional file 1. Restriction enzymes, polynucleotide kinase T4, shrimp-alkaline phosphatase and DNA molecular markers for electrophoresis were all purchased from Thermo Fisher Scientific Inc. (Walthman, MA). DNA T4 ligase was obtained from New England Biolabs (Ipswich, MA). DNA Taq polymerases were purchased from Qiagen (Valencia, CA) and A&A Biotechnology (Gdynia, Poland). PCR reactions were set up in a Veriti® ThermalCycler (Applied Biosystems). PCR was used as a method of obtaining of the final vector construction elements, selecting yeast and bacterial clones, verifying the intermediate constructs completeness, assessing the expression cassette stability. All enzymatic reactions were set up according to the manufacturers’ protocols. All the following steps were conducted with the use of appropriate kit form A&A Biotechnology (Gdynia, Poland): genomic DNA isolation from yeast (Genomic Mini AX yeast kit) and bacterial cells (Genomic Mini kit), PCR products and DNA fragments purification (Clean Up kit), DNA plasmid isolation in small and medium scale (Plasmid Mini kit and Plasmid Midi kit), DNA fragments purification from agarose gel (Gel Out kit).

Schematic representation of the subsequent steps of the expression cassette construction is depicted in (Figure 2). *In silico* design of the genetic construct was preceded with bioinformatic analysis of each DNA sequence element. Due to complexity of the final construct (pYLG1) and resulting lack of unique endonuclease recognition sites, three intermediate constructs were designed (pV1-dhaT, pV1-dhaB2, pV2-dhaB2/ura3). Moreover, introduction of sequences directing the expression cassette integration into a locus of the 28S rDNA gene had to be carried out *ex vivo*, due to the same reason. A 2.2 kb fragment of pBR322 vector, containing *ori* and β-lactamase gene, was used as a platform for construction of the intermediate constructs. The pBR322 fragment was circularized with one of the two linkers, synthesized *in vitro*, containing multiple cloning sites in two restriction variants (see Additional file 1). Followingly, each of the DNA fragments coding for heterologous genes *dhaB1* [GenBank: AAM54728], *dhaB2* [GenBank: AAM54729], *dhaT* [GenBank: AAX12915], *ura3d* [GenBank: YLU40564] with 40 nt upstream and 450 nt downstream fragments, glycerol-induced promoter [GenBank: AJ250328.1] with the first intron, and 150 nt fragment of XPR2-like terminator [GenBank: XM502349] were PCR amplified on the appropriate DNA template. Amplicons were cloned into pGEM-T-Easy (Promega Corporation, MA) vector, and analyzed by endonuclease digestion and sequencing. Subsequently, all pGEM-T-Easy constructs were endonuclease digested, electrophoretically purified and resulting fragments were sequentially cloned into one of the two pBR322-based constructs. As a result, three intermediate vectors were obtained (pV1-dhaT, pV1-dhaB2, pV2-dhaB2/ura3). Their completeness and appropriate orientation of the elements after each cloning were confirmed by PCR and endonuclease digestion. The intermediate vector in each case was composed of two parts: bacterial, containing *ori* and β-lactamase gene, and expression cassette, containing one heterologous gene flanked with regulatory sequences. The intermediate vector pV2-dhaB2/ura3d additionally contained sequence encoding a selective marker gene *ura3d*, with a truncated fragment of promoter. In the next step, intermediate vectors pV1-dhaT and pV1-dhaB1 were endonuclease digested to separate expression cassettes from the bacterial part of the vector. Expression cassettes of the intermediate vectors pV1-dhaT and pV1-dhaB1 were sequentially cloned into an endonuclease digested and dephosphorylated intermediate vector pV2-dhaB2/ura3d. As a result, the final vector pYLG1 was generated. Its completeness and proper orientation of all the elements were verified by PCR amplification and endonuclease digestion. In the next step, the final vector pYLG1 was endonuclease digested in order to separate the expression cassette from the bacterial part. After electrophoresis and purification, the resultant expression cassette was flanked with two sequences complementary to a 28S rDNA gene sequence. As mentioned above, this step was completed *ex vivo*. After phosphorylation and purification, the expression cassette was used for the transformation of competent cells of *Y. lipolytica* A18.

#### Nucleotide sequence analyses – tools and databases

DNA sequences of the heterologous genes, the marker gene and the regulatory sequences were obtained from Genolevures (http://www.genolevures.org) and NCBI (http://www.ncbi.nlm.nih.gov/). Sequences were analyzed in BioEdit package (Tom Hall, Ibis Bioscience) and REviewer© (http://www.fermentas.com/reviewer/app). PCR primers were designed using OligoCalc tool (http://www.basic.northwestern.edu/biotools/oligocalc.html). DNA sequences after sequencing were analyzed with FinchTV (http://www.geospiza.com/Products/finchtv.shtml) and BLAST tool (blastn and blastx) (http://blast.ncbi.nlm.nih.gov/Blast.cgi).

#### RNA isolation and transcript quantification – expression analysis and copy number estimation

RNA from *Y. lipolytica* parental (A18) and recombinant (NCYC3825) strains was isolated according to the protocol provided with Trizol reagent (Life Technologies) after disruption of the yeast cells with glass beads (Sigma-Aldrich) in a Retsch mixer mill (Retsch GmbH, Germany). RNA quantity and quality was analyzed through FA electrophoresis according to [72]. Genomic DNA was eliminated from the samples with DNaseI (Life Technologies). Reverse transcription of RNA to cDNA was carried out with SuperScript III First-Strand Synthesis System for RT-PCR (Life Technologies). Real-time quantitative PCR was carried out with a Real-Time 2xPCR Master Mix SYBR kit A (A&A Biotechnology). Reactions were set up according to the manufacturer’s protocol in Applied Biosystems 7500 apparatus. Real-time PCR primers were designed with Primer Expert Software (Applied Biosystems). The following temperature profile was applied: 94°C 5 min, (94°C 30 sec, annealing temperature according to Primer Expert 15 sec, 72°C – 45 sec ) → 40x, 72°C 5 min, Melt Curve: 94°C 15 sec, 60°C 60 sec, 95°C 30 sec, 60°C 15 sec. Fluorescence from SYBR®Green was measured at the end of elongation step. The reaction was carried out in a comparative mode C_T_ (ΔΔC_T_). Samples were analyzed in triplicate. Raw data analysis was carried out using the software provided by the Applied Biosystems supplier. Depending on the ultimate objective of the real-time PCR analysis, gene expression analysis or expression cassette copy number estimation, different DNA templates and data processing methods were applied, either cDNA and ΔΔC_T_ according to [73], or genomic DNA and modified ΔC_T_ according to [46], respectively. For heterologous genes expression analysis, genomic DNA contamination was monitored through setting up reactions with primers specific to XPR2-like terminator. For an expression cassette copy number estimation, the gene encoding actin was used as an endogenous control, present in a single copy per *Y. lipolytica* haploid genome.

#### Oxidoreductase activity assessment – flow cytometry analysis

BacLight Redox Sensor™ Green Vitality Kit (Thermo Fisher Scientific Inc., Walthman, MA) was used for comparative assessment of oxidoreductase activity in the parental A18 and recombinant NCYC3825 strains. The cells were stained with fluorescent dyes contained in the kit according to the manufacturer’s protocol. Appropriately stained cells were analyzed in BD FACS Aria™III (Becton Dickinson) Cell Sorter. Primary sample line was fitted in the initial 35 μm pore-size filter. The instrument setup (optical alignment), stability and performance tests were carried out using CST system (Cytometer Setup and Tracking; Becton Dickinson). FACSFlow solution (Becton Dickinson) was used as sheath fluid. The configuration of the instrument was as follows: 70 μm nozzle and 70 psi sheath fluid pressure. The analyzed cells were characterized based on the RedoxSensor™ Green fluorescence (FL1 detector), excited with 488 nm blue laser. Threshold line was set on the forward scatter detector (FSC). Data were analyzed with FACS DIVA software (Becton Dickinson). The analysis was preceded by elimination of doublet events, in order to eliminate cellular conglomerates from the analysis. Oxidoreductase activity was determined based on the values of median of green fluorescence. Each sample was analyzed in triplicate.

### Analytical procedures

#### Optical density, dry cellular weight (DCW), lyophilization, total lipid content and fatty acid profile determination

Optical density of culture samples was determined by measurement of absorbance at 600 nm wavelength with Analytik Jena Spectrophotometer and WinASPEKT Software. Dry cellular weight (DCW) was determined through a gravimetric method, after drying the yeast biomass in 105°C until constant mass was reached. Lyophilization was carried out in vials in Christ Beta lyophilizator (Christ Germany), applying the following parameters: initial freezing: -35°C, drying: condenser -35°C, shelf 15°C, pressure 0.22 mbar, time 40 h. Final drying: shelf 20°C, pressure 0.18 mbar, time 8 h. Lyophilized cells were revived by rehydrating the lyophilizate in YPD medium.

Dried biomass was further used for intracellular lipid accumulation determination, which was carried out according to standard gravimetric procedure [74]. Known amount of lyophilized sample was placed in a porous cellulose thimble which was put into extraction chamber (TECATOR). Samples were then repeatedly treated with heated petroleum ether for extraction of peroxide-free lipids from dry yeast biomass, then, the remaining solvent was evaporated and the lipid fraction was determined gravimetrically after drying to the constant weight. The results were expressed as a percentage of total lipid fraction per g of DCW.

For fatty acid profiles determination lyophilized samples were handled according to the procedure described in [75].

#### HPLC analysis

##### Determination of carbon source utilization and metabolites production

Determination of carbon source utilization and metabolites production was carried out through HPLC analysis. 0.45 μm-filtered culture supernatants were analyzed with an Elite LaChrom (VWR-Hitachi) liquid chromatograph equipped with RI L-2490 detector and a Rezex ROA 300 × 7.80 mm column (Phenomenex). 0,005 N H_2_SO_4_ constituted a mobile phase at a flow rate of 0.6 ml min^-1^, isocratic. The column temperature was 40°C. Quantitative and qualitative identification of compounds was carried out with an external standard method, using peak surface for calculations (EzChrom Elite software).

##### Determination of 2-PE and L-Phe concentration

Determination of 2-PE and L-Phe concentration in culture supernatants was carried out through HPLC analysis. 0.45 μm-filtered culture supernatants were analyzed with an Agilent 1200 liquid chromatograph equipped with a DAD detector (254 nm) and a LiChroCART 125-4 Superspher 100 RP-18e (4 μm; MerckMillipore) column. Gradient elution at a flow rate of 1 mL min^-1^ was performed as follows: 0 min – 5% of component B, 10 min-65% B, 11 min - 100% B, 15 min - 100% B, 18 min - 5% B, 23 min - 0% B. Mobile phase components: A – 0.01 M HCl, B – 80:20 (acetonitrile: 0.025 M HCl). The column temperature was 40°C. Quantitative and qualitative identification of 2-PE and L-Phe was carried out with an external standard method, using peak surface for calculations.

#### GC/MS analysis

Identification of 2-PE in the culture supernatants was carried out through GC/MS analysis. Sample preparation: 0.45 μm-filtered culture supernatants were transferred onto activated SPE-C18 cartridge (500 mg per 3 mL, TermoScientific). Extraction was carried out with a vacuum SPE station, at a flow rate of 2 mL min^-1^. Following extraction, the column was washed with 20 mL of deionized water. The non-polar fraction was eluted with 5 mL of pentane: dichloromethane mix at a ratio of 2:1 (v/v), and dried with Na_2_SO_4_. The extract was concentrated to 500 μL and analyzed using GC/MS. GC/MS analysis was carried out with an Agilent 7890A gas chromatograph coupled to a single quadrupole 5975C VL MSD, and equipped with a Supelcowax capillary GC column (60 m × 0.2 mm × 0.2 μm; Sigma Aldrich). Mobile phase: helium at a flow rate of 0.8 mL min^-1^. Inlet port temperature 290°C. Oven program: 1 min: 40°C followed by 8°C per min to 180°C for 1 min, 1 min: 200°C, 20 min: 240°C. Mass detector parameters: transfer line 230°C, scan mode in a range of 33 - 488 m/z, electron impact ionization at 70 eV.

## Competing interests

The authors declare that they have no competing interests.

## Author’s contributions

EC participated in creating the concept of the study, carried out laboratory work and drafted the manuscript. WG participated in creating the concept of the study, coordinated undertakings and approved the manuscript. Both authors read and approved the final manuscript.

## Supplementary Material

Additional file 1: Table S1 PCR primers, real-time PCR primers and linkers used in this study. Click here for file
