# Supplementary material for: A novel multigene expression construct for modification of glycerol metabolism in Yarrowia lipolytica
Source: Microb Cell Fact. 2013 Nov 4;12:102. doi: 10.1186/1475-2859-12-102 (PMC3827991; doi:10.1186/1475-2859-12-102)
Supplement: Additional file 1: Table S1 — PCR primers, real-time PCR primers and linkers used in this study. [file 1475-2859-12-102-S1.docx]

Additional file 1

Tab.S.1

| Primer | Nucleotide sequence | Additional information |
| --- | --- | --- |
| PCR primers | | |
| *dhaB1* F (SalI) | aa GTCGAC atgataagtaaaggatttagtac | amplification of *dhaB1* |
| *dhaB1* R (BglII) | aa AGATCT ttacataacatgttcagttcttg |  |
| *dhaB2* F (BamHI) | aa GGATCC atgagtaagg agataaaagg | amplification of *dhaB2* |
| *dhaB2* R (EcoRV) | aa GATATC ttactcagctccaattgtgcacgg |  |
| *dhaT* F (SalI) | AAG TCG ACA ATG AGC GAC CGC ATG TAT GAC | amplification of *dhaT* |
| *dhaT* R (SacI) | AAG AGC TCT CAG AAT GCC TGG CGG AAG ATG |  |
| P G3Pdh F (EcoRI, XbaI) | aa GAATTC TCTAGA GCCGCACTCGTGCACCCCAAGC | amplification of G3Pdh promoter, three restriction variants |
| P G3Pdh F (EcoRI, MluI) | aa GAATTC ACGCGT GCCGCACTCGTGCACCCCAAGC |  |
| P G3Pdh F (KpnI) | aa GGTACC GCCGCACTCGTGCACCCCAAGC |  |
| P G3Pdh R (SalI) | aa GTCGAC CTGATGTTAGTATGGTAAGCGG |  |
| P G3Pdh R (BamHI) | aa GGATCC CTGATGTTAGTATGGTAAGCGG |  |
| T XPR2-like F (SacI) | aa GAGCTC AGGTTATAAAACTTATTGTC | amplification of XPR2-like terminator, three restriction variants |
| T XPR2-like F (BglII) | aa AGATCT AGGTTATAAAACTTATTGTC |  |
| T XPR2-like F (EcoRV) | aa GATATC AGGTTATAAAACTTATTGTC |  |
| T XPR2-like R (XhoI, XbaI) | aa CTCGAG TCTAGA GATGTCTATTGCAAAGTAGT |  |
| T XPR2-like R (XhoI, MluI) | aa CTCGAG ACGCGT GATGTCTATTGC AAAGTAGT |  |
| *ura3* F (PvuII) | aa CAGCTG CTGCAGACTAAATTTATTTCAGTCTCC | amplification of *ura3d* |
| *ura3* R (NotI) | aa GCGGCCGC GTCGACAAAGGCCTGTTTCTCGG |  |
| pBR322 linker F | CGCTTACAGACAAGCTGTGACCGTCTCC | identification of ligated linkers |
| pBR322 linker R | GGCGTATCACGAGGCCCTTTCGTCTTCAAG |  |
| centr*dhaB1* F | gctaaatctttctatgaagcatg | sequencing the central region of *dhaB1* |
| centr*dhaB1* R | gaatgctgaatcatgccatcc |  |
| rDNA 1 F | CGGGTCCGGCTGCCAGTTGCCCAG | amplification of rDNA L |
| rDNA 1 R (KpnI) | CGGAGTCAACACCCTGGAATTAGTTTG |  |
| rDNA 2 F (NotI) | aaGCGGCCGCGATTCTGCCAAGCCCGTTCCCTTG | amplification of rDNA R |
| rDNA 2 R | AGACACTGCGTCGCTCCGTCCACATCATCAAC |  |
| real-time PCR primers | | |
| r-t *dhaB1* F | AGGAAATCCGGACAACCATTAG |  |
| r-t *dhaB1* R | TTACAAGCTGCAGTAGGTCCAGAT |  |
| r-t *dhaB2* F | GATGGGCCTGGAATAAGAACTATAGT |  |
| r-t *dhaB2* R | GGTTTAATATCTTGGGATTCTGGATTA |  |
| r-t *dhaT* F | GGACCGCCAGCGAAGTC |  |
| r-t *dhaT* R | ATGGAGACGGAAGGCAGGTT |  |
| r-t *ura3* F | GCGACTCTGAGGACTGGCTTA |  |
| r-t *ura3* R | AGCGTCTCCCTTGTCGTCAA |  |
| r-t actin F | CGAGCGAATGCACAAGGA |  |
| r-t actin R | GAGCGGTGATCTTGACCTTGA |  |
| Linkers for the pV1 and pV2 vectors construction | | endonuclease recognition sites |
| LINKER 1/1 | aaagaattcaagcttgacgtcgtcgacggatccgatatcagatctggtaccgcggccgcgagctccagctgcgatcgtctagaacgcgttcctggaaaa | EcoRI, HindIII, AatII, Sali, BamHI, EcoRV, BglII, KpnI, NotI, SacI, PvuII, PvuI, XbaI, MluI, PfoI |
| LINKER 1/2 | ttttccaggaacgcgttctagacgatcgcagctggagctcgcggccgcggtaccagatctgatatcggatccgtcgacgacgtcaagcttgaattcttt |  |
| LINKER 2/1 | aaagaattcgtcgacggtaccgagctcggatccctcgaggatatcagatcttctagagacgtcacgcgtgattccagctgcgatcggcggccgcctgcagtcctggaaaa | EcoRI, Sali, KpnI, SacI, BamHI, XhoI, EcoRV, BglII, XbaI, AatII, MluI, Hindi, PvuII, Psui, NotI, PstI, PfoI |
| LINKER 2/2 | ttttccaggactgcaggcggccgccgatcgcagctggaatcacgcgtgacgtctctagaagatctgatatcctcgagggatccgagctcggtaccgtcgacgaattcttt |  |
